# Adhesion and spreading of osteoblast-like cells on surfaces coated with laminin-derived bioactive core peptides

**DOI:** 10.1016/j.dib.2015.09.032

**Published:** 2015-10-09

**Authors:** In-Sung Yeo, Seung-Ki Min, Hyun Ki Kang, Taek-Ka Kwon, Sung Youn Jung, Byung-Moo Min

**Affiliations:** a Department of Prosthodontics, Seoul National University School of Dentistry, Seoul, Republic of Korea; b Department of Oral and Maxillofacial Surgery, Seoul National University School of Dentistry, Seoul, Republic of Korea; c Department of Oral Biochemistry and Program in Cancer and Developmental Biology and Dental Research Institute, Seoul National University School of Dentistry, Seoul, Republic of Korea; d Department of Dentistry, St. Vincent Hospital, Catholic University of Korea, Suwon, Republic of Korea

## Abstract

Functional peptides are attractive as novel therapeutic reagents because their amino acid sequences are flexible in adopting and mimicking the local functional features of proteins. These peptides are of low molecular weight, synthetically versatile and inexpensive to produce, suggesting that they can be used as drug targeting, potent, stable and bioavailable agents. A short bioactive peptide is expected to be more beneficial in regenerative medicine than an entire protein because of the lower antigenicity of short amino acid sequences. We detected core peptides from human laminin that are involved in adhesion and spreading, which are the first steps of various cells including osteogenic cells, in becoming functional. In this experiment, we detected adhesion and spreading of osteoblast-like cells seeded on the core peptide-coated surface. These *in vitro* data are related to the research article, entitled “Identification of a bioactive core sequence from human laminin and its applicability to tissue engineering” (Yeo et al., 2015) [1].

**Specifications Table**

**Table:** 

Subject area	Biology
More specific subject area	Biomaterial, Biointerface
Type of data	Image (light and electron microscopy) and graphic
How data were acquired	Absorbance, image analysis software (Image-Pro Plus ver. 4.5; Media Cybernetics, Silver Spring, MD, USA), field-emission scanning electron microscopy (S-4700; Hitachi, Tokyo, Japan)
Data format	Analyzed
Experimental factors	Human osteosarcoma and MG-63 cells were cultured in Dulbecco׳s modified Eagle׳s medium supplemented with 10% fetal bovine serum. The cells were seeded on 24-well culture plates or Ti discs coated with the human laminin-derived core peptide. The cells were incubated at 37 °C for 1 h for the adhesion assay or for 3 h for the spreading assay.
Experimental features	Absorbance was measured at 570 nm with a microplate reader for the adhesion assay, and spread cell area was calculated by image analysis for the spreading assay.
Data source location	Seoul, Republic of Korea
Data accessibility	Within this article

**Value of the data**

• The data provide applications of the PPFEGCIWN bioactive core sequence to biological environments in order to upregulate osteogenesis.
• The data inform future studies of bioorganic molecules coated on titanium (Ti) surfaces for desired biologic responses.
• The data are a reference for researchers evaluating the affinity of bone forming cells to bioactive surfaces.

## 1 Data

The affinity of osteogenic cells was evaluated for the bioactive core sequence derived from the human laminin α2 chain, PPFEGCIWN [1]. Here, two types of osteoblast-like cells were seeded on this peptide. Also, those cells were tested for activity on the core sequence-coated titanium (Ti) surface, since the modification of Ti implant surface, including the coating of a bioactive material, is a very important issue to enhance bone healing process around the surface in the field of biomedical regeneration and engineering [2]. Data were collected using well-established protocols to evaluate cell adhesion and spreading *in vitro*.

## 2 Experimental design, and materials and methods

### 2.1 Cells and peptides

The human osteosarcoma cell lines HOS and MG-63 were purchased from the American Type Culture Collection (Rockville, MD, USA) and cultured in Dulbecco׳s modified Eagle׳s medium (Gibco BRL, Carlsbad, CA, USA) supplemented with 10% fetal bovine serum. All peptides were synthesized using the 9-fluorenylmethoxycarbonyl (Fmoc)-based solid-phase method with a C-terminal amide and the Pioneer peptide synthesizer (Applied Biosystems, Foster City, CA, USA). The peptides were purified and characterized at Peptron (Daejeon, Korea). The purity of all peptides was >95% as determined by high-performance liquid chromatography.

### 2.2 Cell adhesion and spreading assays

**Fig. 1:**
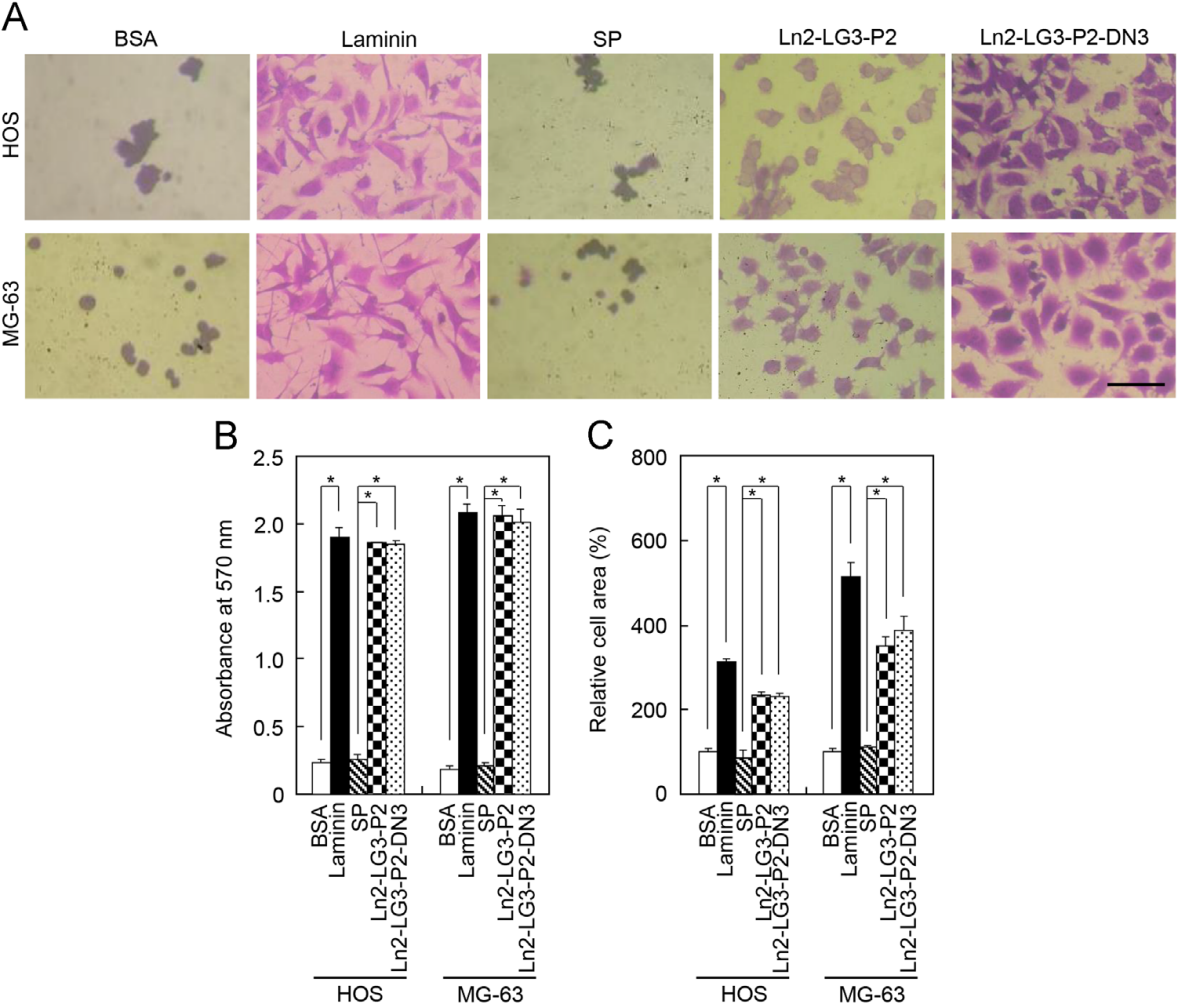
Cell adhesion and spreading of osteoblast-like cells seeded on culture plates treated with laminin, scrambled peptide (SP), Ln2-LG3-P2, and Ln2-LG3-P2-DN3. (A) Photographs of osteoblast-like HOS and MG-63 cells adhering to culture plates treated with 1% bovine serum albumin (BSA), laminin (1.3 μg/cm^2^), SP, Ln2-LG3-P2, and Ln2-LG3-P2-DN3 (13.2 μg/cm^2^) for 3 h. Bar=100 μm. (B) and (C) Adhesion (B) and spreading (C) of osteoblast-like HOS and MG-63 cells seeded on plates treated with BSA, laminin, and synthetic peptides for 1 h (B) or 3 h (C). Data are mean±standard deviation (*n*=4). ^*^p<0.01.

Twenty-four-well plates (Nunc, Rockilde, Denmark) were coated with the human placental laminin (1.3 μg/cm^2^; Sigma-Aldrich, St. Louis, MO, USA), scrambled peptide, RNIPPFEGCIWN bioactive dodecamer (13.2 μg/cm^2^; Ln2-LG3-P2) from the human laminin α2 chain, or the PPFEGCIWN nonamer (13.2 μg/cm^2^; Ln2-LG3-P2-DN3) after drying for 24 h at room temperature and washing with phosphate-buffered saline (PBS). These concentrations were the lowest at which the maximum level of attachment activity of osteogenic cells was determined from dose-response curves [3]. Cell adhesion and spreading were assayed as described previously [4]. Briefly, HOS and MG-63 cells were detached by trypsin digestion, 500 μl of each cell suspension containing 1×10^5^ cells was placed on each well of a 24-well plate coated with peptide, and the cells were allowed to settle/adhere for 1 h for the adhesion test and 3 h for the spreading evaluation at 37 °C in a 5% CO_2_ atmosphere. Both loosely adherent and unbound cells were removed by aspiration, and the wells were washed once with PBS. The remaining bound cells were fixed with 10% formalin in PBS for 15 min and stained with 0.5% crystal violet for 1 h. The wells were gently rinsed with double-distilled water (DDW) three times and photographed using an Olympus BX51 microscope at 100×magnification (Olympus, Tokyo, Japan). Representative images are shown in Fig. 1A. The contents of each well were solubilized in 2% sodium dodecyl sulfate (SDS) for 5 min for the adhesion assay, and absorbance was measured at 570 nm with a microplate reader (Bio-Rad, Hercules, CA, USA). The area of spreading cells was determined using a computer equipped with Image-Pro plus software (Media Cybermetics, Silver Spring, MD, USA). At least 200 cells were examined on each occasion. Data were analyzed using one-way analysis of variance (ANOVA) and Scheffe׳s post-hoc test. A *p*-value of <0.05 was considered significant. Analyses were performed using the STATISTICA 6.0 software package (StatSoft, Tulsa, OK, USA). The data are provided in Fig. 1B for the adhesion assay and Fig. 1C for the cell spreading assay.

### 2.3 Scanning electron microscopy (SEM) of osteoblast-like cells on Ti surfaces coated with bioactive peptides

An SEM was used to examine cell attachment and spreading of HOS and MG-63 cells cultured on Ti surfaces. One Ti disk (20 mm in diameter and 0.5 mm in thickness) was placed per well of a 12-well plate (Nunc). The culture plates containing the Ti disks were coated with 1% bovine serum albumin (BSA), human placental laminin (1.4 μg/cm^2^), or synthetic peptides (14.3 μg/cm^2^), and dried for 24 h at room temperature, blocked with 1% heat-inactivated BSA for 1 h at 37 °C, washed with PBS, and seeded with 1 ml of a cell suspension containing 2×10^5^ cells/ml. The cultures were incubated for 1 h at 37 °C in 5% CO_2_. The loosely adherent or unbound cells were removed by aspiration, wells were washed once with PBS, and the remaining bound cells were fixed with 10% formalin in PBS for 15 min and stained with 0.5% crystal violet for 1 h. The Ti disks were transferred to new 12-well plates and gently rinsed three times with DDW. Next, the contents of each plate were solubilized in 2% SDS for 5 min, and absorbance was measured at 570 nm with a microplate reader (Bio-Rad).

**Fig. 2:**
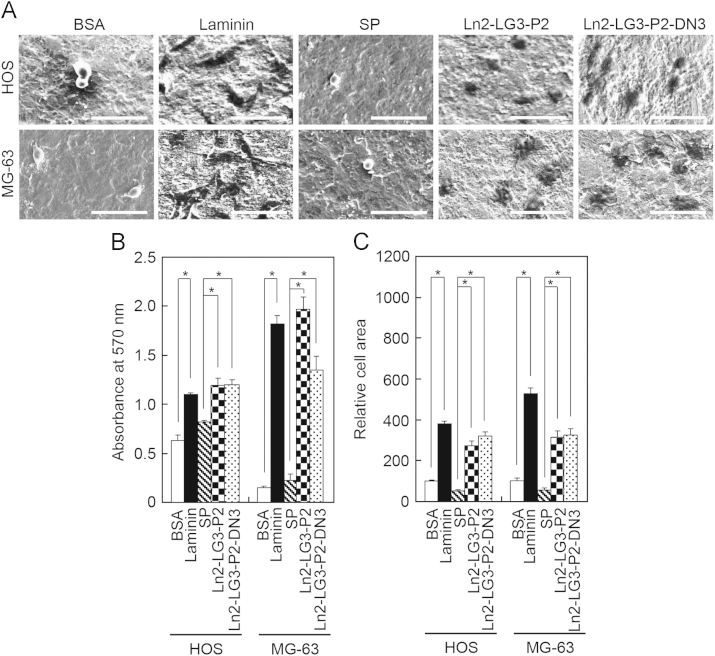
Cell adhesion and spreading of osteoblast-like cells plated on modified Ti surfaces. Scanning electron micrographs (A) and levels of cell adhesion (B) and spreading (C) of osteoblast-like HOS and MG-63 cells plated for 1 h (B) and 3 h (A) and (C) on 1% bovine serum albumin (BSA)-, laminin (1.4 μg/cm^2^)-, scrambled peptide (SP)-, Ln2-LG3-P2-, and Ln2-LG3-P2-DN3 (14.3 μg/cm^2^)-treated pure Ti surfaces. Data are mean±standard deviation (*n*=4). Bars=50 μm. ^*^p<0.01.

Cells (1×10^5^ cells/ml) were added to the substrate-coated Ti disks and incubated for 3 h at 37 °C for the spreading assay. After the incubation, unattached cells were removed by rinsing the plates once with PBS. The attached cells were fixed with 4% paraformaldehyde in PBS for 15 min and the fixative was aspirated. After washing in buffer, the Ti disks were dehydrated through a graded ethanol series. After critical point drying (HCP-2; Hitachi, Tokyo, Japan), the samples were sputter coated with Au/Pd using an SEM coating system (Quorum Q150T-S; Quorum Technologies, West Sussex, UK), and the probes were examined by field emission-scanning electron microscopy (FE-SEM; Hitachi S-4700; Hitachi) at 15 kV. To ensure a representative count, each Ti disk was divided into quarters and one field in each quarter was photographed using an FE-SEM and counted. Representative images of each group are shown in Fig. 2A. Data were analyzed using ANOVA and Scheffe׳s post-hoc test. A *p*-value of <0.05 was considered significant. The analyses were performed using the STATISTICA 6.0 software package (StatSoft). The data are provided in Fig. 2B for the adhesion assay and in Fig. 2C for the spreading assay.
